# Evaluation of the reliability and validity of the Persian version of the Reported and Intended Behaviour Scale, a mental health stigma-related behaviour measure

**DOI:** 10.3389/fpsyt.2025.1553002

**Published:** 2025-05-06

**Authors:** Sima Garmehi, Bahareh Hakimi, Fateme Farhoudi, Ehsan Abedini

**Affiliations:** ^1^ Department of Psychiatry, School of Medicine, Imam Reza Hospital, North Khorasan University of Medical Sciences, Bojnourd, North Khorasan, Iran; ^2^ Psychiatry and Behavioral Sciences Research Center, Mazandaran University of Medical Sciences, Sari, Iran; ^3^ Psychiatry and Psychology Research Center, Tehran University of Medical Sciences, Tehran, Iran; ^4^ Research in Health Sciences, Research and Technology Vice-Chancellor, Mazandaran University of Medical Sciences, Sari, Iran

**Keywords:** behaviour, stigma, mental health, psychometric, Persian

## Abstract

**Background:**

Several instruments have been developed to measure stigma associated with mental disorders. The Reported and Intended Behaviour Scale (RIBS) is one of the validated questionnaires to assess the presence of stigma and discrimination against people with mental illness in the general population. It consists of eight items, divided into two subscales that measure reported and intended stigmatizing behaviours. This study aimed to translate and validate the RIBS in Persian.

**Methods:**

We translated the RIBS questionnaire from English into Persian (RIBS-P) and back-translated it into English. Thereafter, ten psychiatrists evaluated its face validity. Between 2020 and 2021, 384 Persian-speaking adults (aged 18–60) residing in various cities across Iran, with internet access, participated in the study. Exploratory factor analyses were performed to determine the construct validity. The reliability of the questionnaire was assessed using internal consistency (Cronbach’s alpha coefficient) and test-retest methods.

**Results:**

Most of the participants were 28-37 (53.9%), female (64.6%), married (51.6%), unemployed (67.7%) and educated (100%). The total Cronbach’s alpha coefficient for the RIBS-P questionnaire was high (0.732) and The test-retest results showed no significant difference. Factor analysis was used for construct validity and resulted in the extraction of one factor, the mean, standard deviation, and Cronbach’s alpha coefficient for the extracted factor were 3.25, 1.02 and 0.807. The items with the highest correlation with the extracted factor from the exploratory analysis were identified (>0.6). Age (0.00000010701), working status (0.00000010833), and education (0.00000010329) had a significant relationship with stigmatisation behaviour.

**Conclusion:**

The findings indicate that the Persian version of the RIBS is valid and reliable for assessing stigmatizing behaviours among Iranians.

## 1 Introduction

Mental illness is one of the most common health problems in the world (1, 2). Although there are several treatments for psychiatric disorders, there is still a gap between getting sick and asking for help (3). Stigma seems to be one of the barriers to this gap (4, 5). Stigma was expressed as a universal term that includes ignorance, prejudice and discrimination (6). There are several types of mental health-related stigma, including self-stigma, public stigma, professional stigma and institutional stigma. (7–12). The stigma generally results from irrational generalizations, ignorance and fear of those who are different from others (13–15). Hayward and Bright investigated the root causes of stigma toward patients with mental illness and mentioned four public perceptions: 1) Fear of dangerousness, 2) Poor prognosis of mental illness, 3) Attribution of responsibility, and 4) Disruption of social interaction (16). People avoid psychiatric patients in public because they believe that they are dangerous, rare, untreatable, unpredictable and they are responsible for their condition. (17). These discriminatory behaviours cause a gap between the general public and mentally ill patients and create barriers to social interactions, access to help and overall well-being for these patients. By avoiding discriminatory behaviours, the general public can play an important role in the rehabilitation of patients with mental disorders through positive programs in social media, campaigns, and education. In this regard, international organizations such as the World Health Organization (WHO) and the United Nations (UN) strongly recommend systematic and multifaceted interventions to tackle stigma against people with mental illness (18, 19).

Various instruments have been developed to assess specific components of stigma, including the Discrimination and Stigma Scale (DISC) (20), the Inventory of Subjective Stigma Experiences (ISE) (21), and the Consumer Experiences of Stigma Questionnaire (CESQ) (22). In Iran many stigma-related scales were validated to evaluate the dimensions of stigma: OMSHC questionnaire (23), Social distance items(SDI), Perceived dangerousness of mental patient items(PDMPI) (24), Community attitudes towards the mentally ill (CAMI) (25), Stigma of Suicide Scale (SOSS) (26).

The Reported and Intended Behaviour Scale (RIBS) is a valid questionnaire for analysing the existence of reported and intended stigmatizing behaviours against people with mental illness and this scale measures public stigma towards mentally ill patients. RIBS has been used in several local and national studies on stigma, particularly in the UK (27). Considering that the RIBS is a short and easy scale for assessing public behaviour against mentally ill patients and it can estimate stigmatizing behaviour in future (27) and due to the lack of scales for measuring discriminatory behaviours in present and estimating this behaviour in future in Iran, the main purpose of this study was to develop a Persian version of the RIBS questionnaire and to evaluate its validity and reliability. This study could provide a valid and reliable tool to evaluate the reported and intended stigmatizing behaviour among Persian language societies. Such an evaluation might lead to future programs aimed at reducing the public stigma against patients suffering from mental disorders.

## 2 Material and methods

### 2.1 Participants

We developed an online questionnaire and used convenience/accidental sampling to recruit. A total of 384 Persian-speaking adults (aged 18–60 years) residing in various cities across Iran, with internet access, participated in the study between 2020 and 2021. Exclusion criteria included low literacy levels and unwillingness to sign the consent form.

### 2.2 Ethical considerations

The study was reviewed and approved by the Ethical Committee of Mashhad University of Medical Sciences (Ethical code: IR.MAMS.MEDICAL.REC.1399.570). The participants provided their written informed consent to participate in this study.

### 2.3 Reported and Intended Behaviour Scale (RIBS)

This tool was developed by Dr (28) to assess stigma-related behaviour in the general public. The RIBS questionnaire compromises two subscales and a total of eight items. The first subscale, consisting of four items, focuses on reported behaviours (in present or past experiences) in the areas of living, working, living nearby, or having a relationship with a person who has a mental health problem. Each item scores 1 for “Yes” and 0 for “No” or “I do not know”; higher scores indicate more engagement with people with mental health disorders in the past or present. The second subscale, consisting of four items, evaluates the intention to interact with individuals who have mental health problems in the same areas of living, working, living nearby, or having a relationship with a person who has a mental health problem in the future. Participants score items in the second subscale on a 5-point Likert scale ranging from “Strongly Agree” to “Strongly Disagree”. The minimum score is 1 and the maximum is 5; higher scores indicate more stigmatised behaviour towards patients suffering from mental illnesses (29; Garcia et al., 2017).

### 2.4 Translation process and study design

First, permission to translate the questionnaire into Persian was obtained through emails from Dr. Sara Evans-Lacko. The RIBS questionnaire was translated into Persian (RIBS-P) by an expert fluent in both Persian and English. The Persian version was then back-translated into English by two psychiatrists with expertise in the English language, and the two English versions were compared for consistency. To evaluate the validity of the test, face validity was employed. The translated scale was reviewed by 10 psychiatrists, who provided feedback on whether each question of the scale was “simple”, “clear” and “relevant “by questions like: “Is the statement clear?”; “Do you think it could be rewritten more clearly?” and “Did you find it difficult to choose a suitable response for the statement? Necessary corrections were made based on their input.

Once the final version was prepared, the questionnaire, along with the filling instructions, was made available to participants via email and other social network platforms. All the participants were assigned the written formed consent with a questionnaire and send it picture to us. Finally, after receiving all the questionnaires, the validation process was conducted.

### 2.5 Reliability/validity and statistical methods

The validity of the RIBS-P was assessed by face validity and construct validity methods. Exploratory factor analysis was conducted to evaluate construct validity. The reliability of the questionnaire was determined through internal consistency (using Cronbach’s alpha coefficient) and test-retest methods. To apply the test-retest method, 30 participants completed the questionnaire two weeks apart. Data analysis was performed using SPSS 17 and Amos 16 software.

## 3 Results

### 3.1 Demographic characteristics

Most participants were 28 to 37 years old (53.9%), 64.6% participants were female and 35.4% were male. Additionally, 51.6% were married and 48.4% were single. A majority (67.7%) of the participants were unemployed. Most participants had a high level of education: (9.2%) had a Diploma, (7.2%) had an Associate degree, (26.8%) had a Bachelor’s degree and (37.2%) had a Doctoral degree (**Table 1**).

**Table 1:** Participant characteristics.

Characteristic			P-value
Age, years	N (%)	Mean (std)	0.033
<18	27 (7.0)	2.78 (.56)	
18-27	91 (23.7)	3.09 (.57)	
28-37	207 (53.9)	3.74 (.51)	
38-47	47 (12.2)	2.82 (.61)	
48≤	12 (3.1)	2.86 (.64)	
Gender			0.370
Male	136 (35.4)	2.80	
Female	248 (64.6)	2.84	
Marital status			0.986
Married	198 (51.6)	2.82 (.59)	
Single	186 (48.4)	2.83 (.59)	
Working status			0.031
Full-time	124 (32.3)	3.19 (.54)	
Not working	260 (67.7)	2.80 (.62)	
Education			0.026
Diploma	37 (9.2)	2.78 (.56)	
Associate degree	28 (7.2)	3.09 (.57)	
Bachelor’s degree	103 (26.8)	2.74 (.51)	
Master’s degree	73 (19)	3.65 (.61)	
Doctoral Degree	143 (37.2)	2.86 (.64)	

### 3.2 RIBS-P response frequencies

**Table 2** shows the distribution of scores for RIBS-P items in the “reported behaviours” and “intended behaviours” subscales. Responses to the “reported behaviours” subscale indicate moderate contact with individuals with mental illness, ranging from 42.7% to 53.4%. In the “intended behaviours” subscale, most participants strongly disagreed with the statements, with 55.5% unwillingness to live with, 43.8% unwilling to work with, and 44.8% unwilling to live near someone with a mental health problem. Interestingly, 33.1% of responses to the item “willingness to continue a relationship with a friend who developed mental health problems” shifted towards agreement.

**Table 2:** Response frequencies (N = 384).

RIBS item	Yes n (%)	No n (%)	Don’t know n (%)			
Are you currently living with, or have you ever lived with, someone with a mental health problem?	185 (48.2)	176 (45.8)	23 (6.0)			
Are you currently working with, or have you ever worked with, someone with a mental health problem?	164 (42.7)	179 (46.6)	41 (10.7)			
Do you currently have, or have you ever had, a neighbour with a mental health problem?	185 (48.2)	176 (45.8)	23 (6.0)			
Do you currently have, or have you ever had, a a close friend with a mental health problem?	205 (53.4)	152 (39.6)	27 (7.0)			
	Strongly agree n (%)	Slightly agree n (%)	Neither agree nor disagree n (%)	Disagree slightly n (%)	Strongly disagree n (%)	Don’t know n (%)
In the future, I would be willing to live with someone with a mental health problem	7 (1.8)	19 (4.9)	76 (19.8)	56 (14.6)	213 (55.5)	13 (3.4)
In the future, I would be willing to work with someone with a mental health problem	17 (4.4)	42 (10.9)	97 (25.3)	54 (14.1)	168 (43.8)	6 (1.6)
In the future, I would be willing to live nearby to someone with a mental health problem	15 (3.9)	31 (8.1)	89 (23.2)	56 (14.6)	172 (44.8)	21 (5.5)
In the future, I would be willing to continue a relationship with a friend who developed a mental health problem	85 (22.1)	127 (33.1)	57 (14.8)	33 (8.6)	67 (17.4)	15 (3.9)

### 3.3 Validity assessment

**Figure 1:**
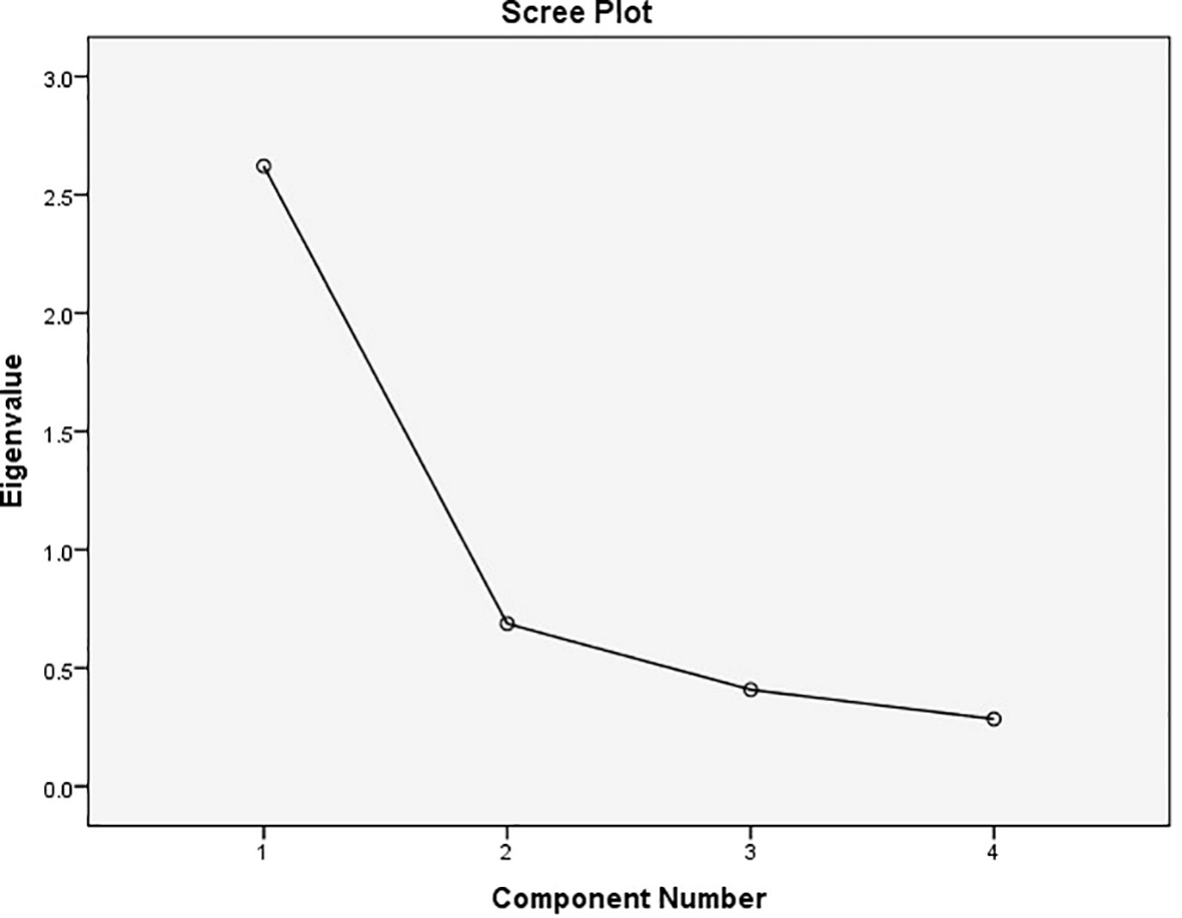
Domain slope diagram of test variables.

The results demonstrated that the Kaiser-Mayer-Olkin (KMO) index was 0.778 (>0.6), indicating that the data were adequate for factor analysis. Additionally, the significance level of Bartlett’s test of sphericity was less than 5%, suggesting that exploratory factor analysis is appropriate for finding factors and that the data correlation matrix in the community is not zero (**Table 3**). Principal component analysis was conducted using the Varimax rotation method. Several experimental rotations were performed to explain the most appropriate factors. Ultimately, based on the slope of the Scree plot diagram, one factor with an Eigenvalue higher than 1 was identified. A single factor was accepted, explaining 65.526% of the total variance, because of the structure validity analysis (**Table 4** and **Figure 1**). The results of repeated exploratory analysis showed that the common values of the research variables (questions) were higher than 0.5 indicating optimal values (**Table 5**). The mean, standard deviation, and Cronbach’s alpha coefficient for the extracted factor were 3.25, 1.02 and 0.807, respectively (**Table 6**). According to **Table 7**, the items with the highest correlation with the extracted factor from the exploratory analysis were identified (>0.6).

**Table 3:** KMO and Bartlett’s tests.

Kaiser-Meyer-Olkin Measure of Sampling Adequacy		0.778
Bartlett Test of Sphericity	Approx. Chi-Square	596.790
	Df	6
	Sig.	0.000

**Table 4:** Total variance explained.

Component	Total	% of Variance	The cumulative variance %
1	2.621	65.526	65.526

**Table 5:** Component matrix.

Items	Component
Item 5	0.663
Item 6	0.773
Item 7	0.731
Item 8	0.534

**Table 6:** Mean, standard deviation and Cronbach’s α coefficient of factor derived from exploratory analysis.

	Mean	Standard deviation	Cronbach’s α coefficient
Item 1	3.25	1.02	0.807

**Table 7:** Questions regarding each factor derived from exploratory analysis.

Variable (question)	Extractive shared values
Item1	0.827
Item2	0.879
Item3	0.855
Item4	0.659

### 3.4 Reliability assessment

The reliability of the questionnaire was assessed using internal consistency (measured by Cronbach’s alpha coefficient) and the test-retest. The test-retest results showed no significant difference, confirming the reliability of the RIBS-P scale. The reliability of the RIBS-P scale was estimated to be 0.732 according to Cronbach’s alpha coefficient (**Table 8**).

**Table 8:** Reliability of the RIBS.

		Test	Retest
RIBS items	Item Mean (s.d.) N=384	Internal Consistency (Cronbach’s α) N=384	Internal Consistency (Cronbach’s α) N=384
Are you currently living with, or have you ever lived with, someone with a mental health problem?	–	0.757	0.761
Are you currently working with, or have you ever worked with, someone with a mental health problem?	–		
Do you currently have, or have you ever had, a neighbour with a mental health problem?	–		
Do you currently have, or have you ever had, a close friend with a mental health problem?	–		
In the future, I would be willing to live with someone with a mental health problem	2.72 (1.08)	0.807	0.813
In the future, I would be willing to work with someone with a mental health problem	3.13 (1.25)		
In the future, I would be willing to live nearby to someone with a mental health problem	2.95 (1.25)		
In the future, I would be willing to continue a relationship with a friend who developed a mental health problem	4.22 (1.51)		
Total		0.732	0.755

## 4 Discussion

The present study was designed to evaluate the validity and reliability of the Persian version of the Reported and Intended Behaviour Scale (RIBS-P). Ten psychiatrists evaluated the face validity of the translated questionnaire, and all items of scale were accepted in RIBS-P without any modification. A sample of 384 adults completed it. After that, exploratory factor analyses were performed to determine the construct validity. The reliability of the questionnaire was assessed using internal consistency (Cronbach’s alpha coefficient) and test-retest methods.

The results of this study demonstrated a significant relationship between stigmatizing behaviours towards patients with mental illnesses with occupational status, age, and education (**Table 9**). In the Japanese version of RIBS, there was just a weak correlation between age and stigmatizing behaviours and more contact with mentally ill patients in the elderly could explain this correlation (29), In the Brazilian version of RIBS there was a high socioeconomic status with less intended stigmatisation behaviours (30). previous research has shown that the belief that mental health problems are a sign of weakness decreases with age, but the belief that mental health problems make a person dangerous and unpredictable increases with ageing (31). It has been demonstrated that early adolescents generally report more positive intended behaviours towards patients with mental illnesses (Mansfield, Humphrey and Patalay, 2020). The results of our study on the relationship between age and stigmatizing behaviours are consistent with other studies, as mentioned. In this study, education and employment were significantly associated with stigmatised behaviours. To explain this significance, we can mention the three dimensions of stigma: knowledge, behaviour, and attitude. Individuals with higher education or those who are employed tend to have greater knowledge about mental disorders. This increased knowledge may contribute to reducing stigmatised attitudes and behaviours among them (6, 32)

**Table 9:** Relationship between Stigma and demographic variables.

Variable	t	F	r	P-Value
Age	–	–	0.425	<0.0001
Gender	0.130	–	–	0.710
Marital status	0.241	–	–	0.080
Working status	2,534	–	–	<0.0001
Education	–	3.302	–	<0.0001

According to the results, Our sample of the Iranian population had a higher level of contact with people with mental disorders (42.7%-53.4%) compared to those in the United Kingdom (17.7%-32.5%), Italy (14.77%-27.07%), Japan (14.7%-39.7%) and the Czech Republic (12.7%-15.3%) (29; Winkler et al., 2015; 33). On the other hand, the Iranian population was less inclined to make contact with patients with mental illnesses in the future (slightly disagree + strongly disagree: 26%-70.1%) compared to the British population (agree + strongly agree: 55.9%-81.9%) (Winkler et al., 2015). This notable difference between the two items of the questionnaire in the Iranian population despite the British population can be attributed to the lack of structured programs to raise knowledge about mental disorders. This insufficient knowledge may lead to negative attitudes and beliefs, causing individuals to perceive those with mental health conditions as unsuitable for living, working, or forming friendships in the future. This observation strongly highlights the need for anti-stigma programs and campaigns (34–36). In contrast to other items, most of our participants (55.2%) were inclined to continue a relationship with a friend who developed a mental health problem in the future, which is consistent with the results of a study in Japan (29). Cultural similarity in Eastern countries and their collective and family-oriented lifestyles could explain this result and could act as a protective factor (37)

The Cronbach’s alpha coefficient for the reliability of the questionnaire was high (0.73). No significant differences were observed between test-retest results, confirming the reliability of the RIBS-P questionnaire. The internal consistency of the second RIBS-P subscale was high and satisfactory, exceeding the minimum threshold of 0.7 (α = 0.807) (38). In the study by Pingani et al. evaluating the validity and reliability of the Italian version of the RIBS questionnaire, the internal consistency of the second subscale was 0.83 (33). Similarly, Yamaguchi et al. (29) reported an internal consistency of 0.83 for the Japanese version of the questionnaire. The results of our study were consistent with these studies.

Our results indicated that the Kaiser-Meyer-Olkin (KMO) Sampling Adequacy Index was 0.778, exceeding the minimum threshold of 0.6, which suggests that the sample size was sufficient for factor analysis (39). Additionally, the significance level of Bartlett’s test of sphericity was less than 5% (40).

To determine the construct validity of the questionnaire, exploratory factor analysis was conducted. This analysis led to the extraction of a single factor, which accounted for 65.52% of the total variance of the test variables. The Cronbach’s alpha coefficient for the extraction factor was 0.807, indicating high reliability (>0.7). Each item demonstrated a strong correlation with the extracted factor, with a value exceeding 0.6.

So far, few studies have evaluated the validity and reliability of various versions of the RIBS questionnaire. Yamaguchi et al. and Pingani et al, employed confirmatory factor analysis to assess validity, with results indicating a good model fit (χ2 = 41.001, df = 19, P = 0.002 and χ2 = 23.60, df = 19, p = 0.21 respectively) (29, 33).

One limitation of the present study was the lack of control over the participants’ place of residence. Cultural differences between urban and rural areas could contribute to variations in attitudes and stigmatizing behaviour and we recommend future studies to address this gap. Additionally, the study could not objectively verify whether participants had a mental illness, which was intended as an exclusion criterion. This limitation may introduce biases in responses when completing the online questionnaire. Another limitation of our study was the predominance of highly educated participants, In future research, it is recommended to select a more varied sample population. In this study we didn’t assess criterion validity for the RIBS questionnaire and including such comparisons would strengthen the validity of the scale for future studies.

## 5 Conclusion

In this study, we tested the psychometric properties of the Persian version of the RIBS questionnaire. The results showed good internal consistency and reasonable test-retest reliability, consistent with findings from other studies. The construct validity assessment, including both exploratory factor analysis, was also found to be appropriate. Therefore, we consider the RIBS-P to be an appropriate and psychometrically robust scale for assessing stigmatizing attitudes and behaviours towards patients with mental health problems in Iranian society.

## Data Availability

The raw data supporting the conclusions of this article will be made available by the authors, without undue reservation.
